# Histiocytosis X of the temporal bone

**DOI:** 10.1016/S1808-8694(15)31010-7

**Published:** 2015-10-19

**Authors:** Lidiane Maria de Brito Macedo Ferreira, João Deodato Diógenes de Carvalho, Sérgio Tadeu Almeida Pereira, Marylane Galvão Tavares

**Affiliations:** a MD, ENT resident - Hospital Geral de Fortaleza; b MD, ENT, head of otorhinolaryngology - Hospital Geral de Fortaleza; c Otorhinolaryngologist, preceptor of the ENT residency program - Hospital Geral de Fortaleza; d MD, ENT resident Hospital Geral de Fortaleza

## INTRODUCTION

Langerhans’ cell histiocytosis (LCH) is characterized by monocytic-macrophagic system cell proliferation. The peak incidence period is between ages 1 and 4^1^. The annual incidence is approximately 3-4 per million^2^, and the disease is twice as common in the male gender.

The clinical picture is variable, but the disease usually affects the head and neck (60% of cases)^3^, including bone lesions, cervical lymphnode enlargement and skin rashes. Temporal bone involvement and otoneurological symptoms are seen in 4 to 25% of cases^4^.

The diagnosis is obtained through a biopsy (inflammatory infiltrate with Langerhans’ cells and the immunohistochemical markers CD1a, S100 and CD101)^2^. Lytic lesions in the temporal bone, seen on computed tomography, increase suspicion of the disease.

Treatment is variable and depends on the type of lesion. The disease may remit spontaneously or cause death regardless of treatment.

## CLINICAL CASE

**Figure 1:**
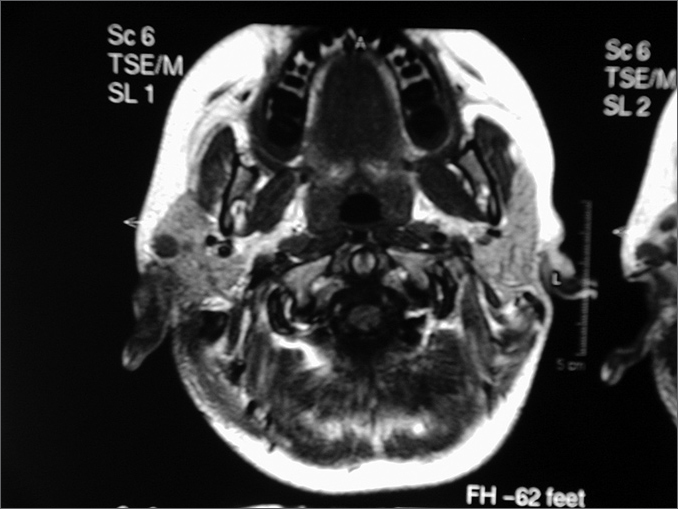
Magnetic resonance imaging of the cranium - presence of lesions in both temporal bones suggesting histiocytosis X.

GMG is an 11-year-old female patient with a history of headaches and right otalgia during the past nine years. On October 2002 she was brought to the Otorhinolaryngology unit when right purulent otorrhea was observed; the physical exam showed a bulging, hyperemic and intact tympanic membrane. Left otoscopy was normal. There was no lymphadenomegaly, and other physical exam findings were within normal limits. She was treated with systemic antibiotics. On September 2003 the patient returned to the outpatient department reporting only slight improvement. Otoscopy showed a slightly bulging and hyperemic tympanic membrane in the right ear. Again the patient was given systemic antibiotics. She reported intermittent improvement. On August 2004 the patient returned with no improvement of her headache and otalgia. A polyp was seen in the external auditory canal of the right ear. Computed tomography of the mastoid revealed opacification and bone destruction to the right; the left mastoid was unaltered. On October 2004 the patient underwent right mastoidectomy for a biopsy. Histopathology and immunohistochemistry confirmed the diagnosis of histiocytosis X. On January 2005 the patient underwent radical mastoidectomy and meatoplasty. Corticosteroid pulse therapy was given in the immediate postoperative period. The bone inventory and abdominal computed tomography were unaltered. On April 2005 the patient presented left otorrhea. Otoscopy showed secretion in the external left auditory canal and a bulging and intact tympanic membrane. The right ear had a meatoplasty. Cranial magnetic resonance imaging revealed a bilateral soft tissue density lesion with no cerebral foci (Figure 1). The clinical decision was to carefully monitor the patient. One and a half year later, the disease is clinically stable and medical observation will be continued.

## DISCUSSION

The treatment of LCH is rather controversial, particularly when dealing with localized forms in the head and neck. Many authors argue that surgery is an invasive procedure in such cases^3^, ^5^. Most papers, however, recommend surgery associated with some other treatment (corticosteroid therapy, chemotherapy^6^ or radiotherapy on focal lesions). Our patient was treated with surgery and corticosteroid therapy, without discarding possible future chemotherapy. For the moment the disease is controlled. The treatment of choice in this case was due to the extension of the lesion, as multicentric lesions required more aggressive therapy.
